# Socioeconomic factors increase the risk of teenage pregnancy: spatial and temporal analysis in a Brazilian municipality

**DOI:** 10.1590/1980-549720240040

**Published:** 2024-07-29

**Authors:** Camila Meireles Fernandes, Gleice Margarete de Souza Conceição, Zilda Pereira da Silva, Fernando Kenji Nampo, Francisco Chiaravalloti

**Affiliations:** I Universidade de São Paulo, School of Public Health, Department of Epidemiology – São Paulo (SP), Brazil.; II Universidade Federal da Integração Latino-Americana, Latin American Institute of Life and Natural Sciences – Foz do Iguaçu (PR), Brazil.

**Keywords:** Spatial analysis, Pregnancy in adolescence, Regression analysis, Temporal distribution, Socioeconomic factors

## Abstract

**Objective:**

To evaluate the distribution of the proportion of teenage mothers (PTM) in time and space and its relationship with socioeconomic indicators and social vulnerability.

**Methods:**

An ecological study was carried out with teenage mothers living in 322 census tracts in Foz do Iguaçu (state of Paraná, Brazil) between 2013 and 2019. Spatial clusters of teenage mothers were identified by spatial scanning and grouped into strata with different prevalence. The association between these strata and the individual social vulnerability of the mothers was evaluated using the Pearson's Chi-square test. Linear regression models were adjusted to evaluate the association between PTM and socioeconomic factors by census tract and temporal trend in PTM in different strata.

**Results:**

We identified five high prevalence clusters in peripheral regions and six with low prevalence in the central region of the municipality. Proportionally, there were more teenage mothers with a worse vulnerability index in the high prevalence stratum than in the low prevalence stratum. Places with worse socioeconomic conditions present higher PTM, a profile that did not change over time. For the increase of one unit in the Brazilian Deprivation Index and proportion of women responsible for the household, the PTM increased, respectively, by 3.8 (95%CI 3.1–4.4) and 0.086% (95%CI 0.03–0.14). There was a reduction in the global PTM in part of the period, which occurred later in the higher prevalence strata, but the proportions were stable again in the last years of study.

**Conclusion:**

Teenage pregnancy is concentrated in regions with worse socioeconomic conditions and greater maternal vulnerability and its behavior over time occurred differently in these areas.

## INTRODUCTION

Adolescence is marked by physiomorphological changes and psychosocial adaptations that are decisive for health and well-being in adult life^1^. Adolescents’ sexual health is part of their development; however, there is lack of information and sexual education, sexual coercion and partner violence, difficult access to contraceptive and abortive methods, and a high pregnancy rate^2^.

Worldwide, maternal mortality in adolescents was 36.8% higher when compared to women aged 20 to 24 years in 2011^3^. Pregnant teenagers have higher rates of depression and greater use of psychoactive substances when compared to adults in studies with data from 2009 to 2014 in Canada^4^. There is a higher prevalence of premature birth, eclampsia, postpartum hemorrhage, low-birth weight newborns, low Apgar scores and anemia, as well as worse mental health in the postpartum period in studies up to 2020^5–7^.

Despite the global decline in teenage pregnancy in recent decades, countries in Sub-Saharan Africa and Latin America and the Caribbean continue having high rates, 104 and 63 births per thousand adolescents, respectively^8^. Brazil showed a reduction in the percentage of live births (LB) of teenage mothers; nevertheless, it presents high values, especially in the North and Northeast regions^9^.

Teenage pregnancy is a challenge, especially in developing countries, where policies on sexual health and education are deficient or scarce^10^. This mostly affects vulnerable individuals, with associated factors being extreme poverty, school dropout, and being the son/daughter of a teenage mother^5,10^. It involves socioeconomic, educational, environmental, and behavioral factors and does not occur uniformly in different regions and populations, in such a way that considering time and space helps to optimize preventive actions^11,12^.

As of 1980, the health sector began to use spatial approaches to analyze events, breaking a predominant pattern of studies only focusing on individuals. Ecological studies, in which the unit of analysis corresponds to a population cluster in time and/or space^13^, help to understand disease patterns and their outcomes^13,14^.

Most of the ecological studies carried out in Brazil spatially analyzing teenage pregnancy had municipalities as the unit of analysis and showed their association with socioeconomic factors, such as education, the Gini Index and the Municipal Human Development Index (MHDI), density of residents per household, *per capita* family income, and proportion of low-income population^11,15–19^. Only three studies were carried out with intra-municipal information^12,16,20^. Among these studies, only a few^11,15,17,19^ considered the spatial dependence of the studied phenomenon, producing more accurate results.

Despite the importance of teenage pregnancy in public health^9,17^, few studies have analyzed it spatially, highlighting a gap in the area, especially when it comes to intra-municipal assessments. Considering that spatial analysis techniques are constantly being updated, new studies on teenage pregnancy with a spatial focus can support decision-making by managers and contribute to the survey of hypotheses based on the importance of territory and socioeconomic factors in the occurrence of the phenomenon.

In this study we aimed to evaluate the distribution of the proportion of teenage mothers (PTM) in time and space and its relationship with socioeconomic indicators and social vulnerability in the municipality of Foz do Iguaçu, between 2013 and 2019.

## METHODS

This is an ecological study.

### Characterization of the study site

Foz do Iguaçu is located in the state of Paraná, Brazil, bordering Paraguay and Argentina. In 2019, it had a population of 258,532 inhabitants and a high *per capita* gross domestic product (GDP) (BRL 60,997.41). The infant mortality rate registered a decrease in the 2023–2019 period (from 14.14 to 10.17 deaths per thousand LB) and the MHDI evolved from medium (0.663) in 2000 to high in 2010 (0.751)^21^. Although these indicators point to its high development in relation to the country, the Brazilian Deprivation Index (*Índice Brasileiro de Privação* – IBP-Cidacs) highlights intra-municipal inequalities. In 2010, the IBP ranged from −3.04 to 2.99 in the different urban census tracts (CT). This is an indicator of socioeconomic status developed based on indicators of income, education, and household condition, using data from the 2010 Census. The higher its value, the greater the deprivation^22^.

### Data

Data on LBs, between January/2013 and December/2019, of mothers living in the municipality, were obtained from the Live Birth Information System (*Sistema de Informação Sobre Nascidos Vivos* – Sinasc). The following Information from live birth certificates (LBC) was considered: age, address, date of birth, number of previous pregnancies, level of education, and start of prenatal care. Records without full addresses were excluded, making geocoding impossible. In multiple pregnancies, only one record was kept to avoid duplication.

In the spatial analysis, the mothers’ addresses were converted into geographic coordinates, using Google My Maps^23^. They were exported to the QGIS software^24^ and superimposed on the map with 322 CT, already considering the exclusion of two peripheral census tracts with no births and a census tract merged with a neighboring one, as it contained only one birth, whose mother was a teenager (thus resulting in a proportion of 100%). Comparing this with the CT in the municipality^25^ enabled to count teenage mothers and the total number of mothers in each census tract, obtaining the PTM, defined as the quotient between the number of teenage mothers and the total number of mothers multiplied by 100. For the temporal analysis, the monthly PTM was estimated.

### Statistical analysis

#### Identification of spatial clusters

To identify spatial clusters with high and low prevalence of teenage pregnancy, the SaTScan scanning software was used, assuming a Poisson distribution^26^. Databases were created with information on the number of teenage mothers, the total number of mothers, and the coordinates of the census tract centroids. The maximum population size of mothers for the scanning window was determined using the Gini index^27^. The p-value was obtained using the Monte Carlo method. The results were aggregated to the census tracts in QGIS, obtaining the map with the clusters and their respective prevalence ratios (PR).

Based on these results, the census tracts were grouped into three strata, comprising areas that were not necessarily contiguous: "PR>1" or "high prevalence," containing census tracts belonging to clusters with PR significantly higher than 1; "PR=1," with those that did not belong to any cluster; "PR<1" or "low prevalence," with those belonging to clusters with PR significantly lower than 1.

#### Association between type of stratum and mother's vulnerability

The World Health Organization (WHO) points out that progress in reducing teenage pregnancy has been slower among vulnerable groups, leading to growing inequality. With data from Sinasc, a vulnerability index for adolescents was developed, consisting of three variables assuming the values 0 or 1: previous pregnancies (0, if none; 1, if one or more), level od education (0, if complete high school or over; 1, if complete elementary school or less), and start of prenatal care (0, if it started in the first trimester; 1, if it started in the second trimester or later). The sum of the values attributed to each mother produced an index ranging from 0 to 3: the higher the value, the greater the mother's social vulnerability. To assess whether this index was associated with the residence stratum, Pearson's χ^2^ test was carried out.

#### Spatial modeling

To assess whether teenage pregnancy was associated with the socioeconomic level of the area of residence, a multiple linear regression model was adjusted, with the PTM as the response variable and, as independent variables, the IBP and the proportion of women responsible for the household (PWRH), obtained based on data from the 2010 Census^28^, according to census tracts. Previously, an exploratory data analysis was carried out. The Global Moran's test was used to evaluate the spatial autocorrelation of the PTM and the residuals of the obtained models. The Queen-type contiguity neighborhood matrix was considered. Initially, a classic linear regression model was adjusted and the spatial dependence of the residuals was identified. Subsequently, the Lagrange multiplier test was used, which showed that the most appropriate spatial autoregressive model (SAR) to represent the spatial autocorrelation not explained by the initial model was that of spatial lag^29^.

#### Temporal modeling

To assess the trend in the proportion of teenage pregnancies, a multiple linear regression model was adjusted. The response variable was the monthly PTM and the explanatory variables were time (months) and residence stratum. To evaluate its assumption of normality, the Kolmogorov-Smirnov test was used. To model changes in trends during the period, segmented regression techniques were used with two inflection points suggested by the data: January/2016 and July/2017. An intercept and slope were estimated for each of the three determined periods (Jan./2013–Jan./2016, Jan./2016–July/2017, July/2017–Dec./2019), according to strata. To assess whether the trend in a given stratum was the same in the three periods, as well as whether the trend in a given period was the same in the three strata, contrasts were constructed, under the general linear hypothesis^30^ involving the corresponding slopes. In situations where they were not significantly different, a single slope was adjusted. Based on the final model, the adjusted PTM at the beginning and at the end of each period were presented, in addition to the variation of these percentages over time, according to strata. To assess whether the model in question was well adjusted, a residual analysis was carried out.

With the exception of the scanning analysis, the others were carried out using the R^31^ software. The significance level of the tests was set at 5%.

This research was approved by the Research Ethics Committee of the Centro Universitário Dinâmica das Cataratas (UDC), CAAE 33186620.7.0000.8527, on July 31, 2020.

## RESULTS

After applying the exclusion criteria, the total number of mothers was 29,507; of these, 4,117 (13.95%) were teenagers. Records referring to twin births (337 mothers, 7.71% teenagers) and those without a full address (809 mothers, 19.03% teenagers) were excluded.

### Spatial clusters and association with mother's vulnerability

**Figure 1:**
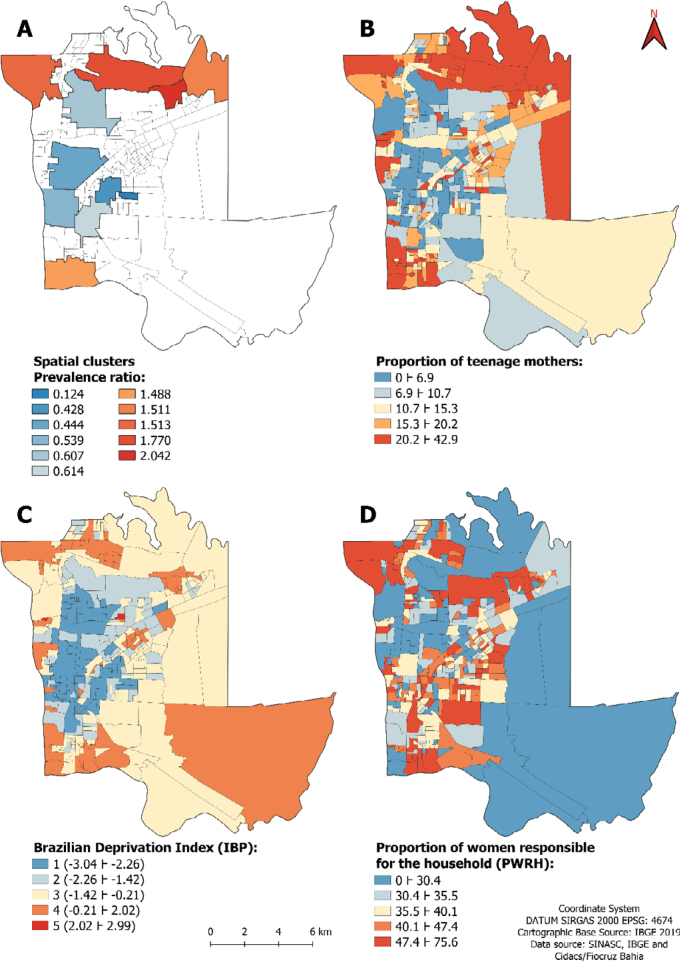
Spatial clusters of high and low prevalence of teenage mothers (A), Proportion of teenage mothers (B), Brazilian Deprivation Index in urban areas (C), and Proportion of women responsible for the household (D), according to census tract. Foz do Iguaçu (PR), Brazil, 2013–2019.

The maximum population size of mothers for the scanning window estimated by the Gini index was 6%. In Figure 1A we show the 11 clusters identified, five with high prevalence (45 census tracts) and six with low prevalence (83 census tracts).

In Table 1 we can observe an association between the vulnerability index and the two strata of census tracts formed by the high and low prevalence clusters (p<0.001). As the vulnerability index increases, the proportion of mothers with PR>1 increases and the proportion of those with PR=1 and PR<1 decreases. Proportionally, there were more teenage mothers with a worse vulnerability index in the high prevalence stratum than in the low prevalence stratum.

**Table 1:** Distribution of teenage mothers according to social vulnerability index and type of spati al cluster. Foz do Iguaçu (PR), Brazil, 2013–2019.

Vulnerability Index	Type of cluster						Total		p-value
	PR>1		PR=1		PR<1				
	n	%	n	%	n	%	n	%	
0	344	24.33	874	61.81	196	13.86	1,414	100	<0.001
1	447	27.63	964	59.58	207	12.79	1,618	100	
2	289	33.45	487	56.37	88	10.19	864	100	
3	77	35.48	114	52.53	26	11.98	217	100	
Total	1,157	28.13	2,439	59.3	517	12.57	4,113	100	

PR: prevalence ratios.

### Spatial modeling

**Figure 2:**
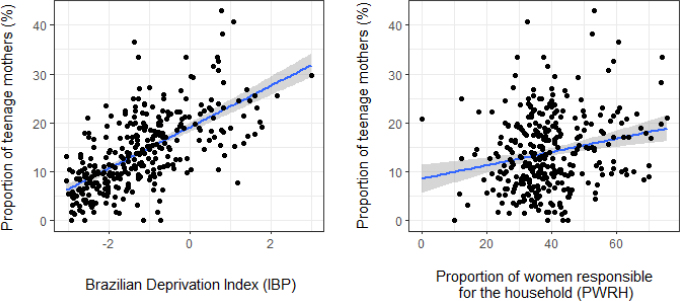
Scatter plot of the proportion of teenage mothers as a function of the Brazilian Deprivation Index and the proportion of women responsible for the household. Foz do Iguaçu (PR), Brazil, 2013–2019.

The Moran's test indicated the existence of spatial autocorrelation in the residuals (I=0.059, p=0.025) of the classic regression model, and a spatial lag model was employed. In this model there was a direct association of the PTM with IBP and the PWRH (Figure 2): with each increase of one unit in IBP, the PTM increases by 3.8% (95%CI 3.1–4.4) and, with each increase in 1% in the PWRH, the PTM increases by 0.086% (95%CI 0.03–0.14), on average. These associations are reflected in Figures 1B, 1C and 1D, respectively with the geographic distribution of the PTM, IBP, and PWRH. The autoregressive spatial coefficient^29^ (Rho=0.14 p=0.055) represented the spatial dependence not explained by the covariates included in the model. The coefficient of determination was 0.44, that is, the model explains 44% of the PTM variability. This model presented a lower Akaike Information Criterion (AIC, 2055.8) than the classic model (2057.4), and its residuals presented normality (p=0.05343, in the Kolmogorov-Smirnov test) and good fit.

### Temporal modeling

**Figure 3:**
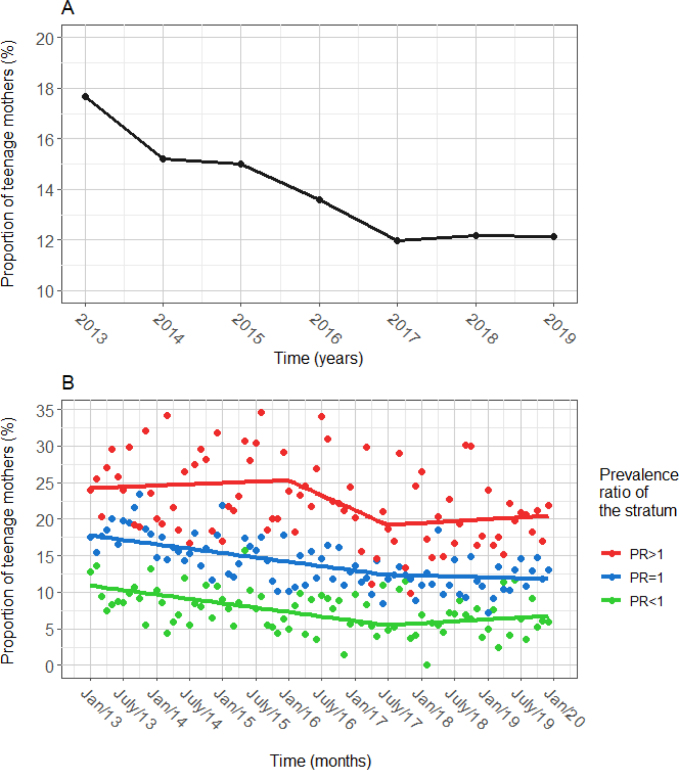
Proportion of teenage mothers over the years (A) and months, according to the prevalence ratio of the strata (B). Foz do Iguaçu (PR), Brazil, 2013–2019.

In Figure 3A we show the PTM over the years, showing a decrease between 2013 and 2019, ranging from 17.67 to 12.13%. In Figure 3B we present the observed (represented by dots) and adjusted (represented by straight lines) PTM over the months, according to the residence stratum. In Table 2 we present the adjusted values for the proportions at the beginning and at the end of each period and the estimated variation over time for each stratum.

**Table 2:** Percentage of teenage mothers adjusted at the beginning and at the end of each period and estimated variation in the proportion over time, according to stratum. Foz do Iguaçu (PR), Brazil, 2013–2019.

Stratum PR	Proportion adjusted in Jan./2013	Variation in the Jan./2013–Jan./2016 period	Proportion adjusted in Jan./2016	Variation in the Jan./2016–July/2017 period	Proportion adjusted in July/2017	Variation in the July/2017–Dec./2019 period	Proportion adjusted in Dec./2019
PR<1	10.9 (9.4; 12.3)	−0.10* ↓ (−0.14; −0.06)	7.3 (6.4; 8.1)	−0.10* ↓ (−0.14; −0.06)	5.5 (4.3; 6.6)	0.04 → (−0.04; 0.12)	6.7 (4.8; 8.7)
PR=1	17.8 (16.3; 19.2)	−0.10* ↓ (−0.14; −0.06)	14.1 (13.2; 15.1)	−0.10* ↓ (−0.14; −0.06)	12.3 (11.1; 13.6)	0.02 → (−0.12; 0.08)	11.8 (9.5; 14.1)
PR>1	24.2 (22.0; 26.4)	0.03* → (−0.06; 0.13)	25.3 (23.4; 27.1)	−0.34* ↓ (−0.49; −0.19)	19.2 (17.6; 20.7)	0.04 → (−0.04; 0.12)	20.4 (18.7; 22.2)

PR: prevalence ratios.

* p<0.001. The arrows indicate the temporal variation in the specific period. The downward arrow indicates a reduction, and the arrow to the right indicates that there was no change in the period.

In the PR<1 and PR=1 strata, the PTM decreased by 0.1% (95%CI 0.06–0.14) per month, until July 2017. In the first, the proportion decreased from 10.9 (in January/2013) to 5.5% (in July/2017). In the second, the proportion decreased from 17.8 (in January/2013) to 12.3% (in July/2017). As of July/2017, the trends were not significant. In December/2019, the adjusted proportions were 6.7 and 11.8% in the PR<1 and PR=1 strata, respectively.

Unlike the other two strata, in PR>1, the proportion only started decreasing in January/2016. From January/2016 to July/2017, there was an estimated drop of 34% (95%CI 19–49) per month, significantly greater than that observed in other strata. As of July 2017, following what happened in the other strata, the decrease ceased and the trend remained stable until the end of the study.

Throughout the period, the adjusted PTM values were highest in the PR>1 stratum, followed by the PR=1 and, finally, the PR<1. In January/2013, the adjusted proportion in the PR>1 stratum was more than double the proportion in the PR<1; in July/2017, this value reached 3.5 times and did not undergo major changes until the end of the study. The residuals from the temporal modeling showed normality (p=0.3525, in the Kolmogorov-Smirnov test) and good fit.

## DISCUSSION

According to the analyses carried out, there is a consistent relationship between the occurrence of teenage pregnancy and socioeconomic indicators and social vulnerability. Places with a higher maternal vulnerability index and worse socioeconomic conditions showed higher PTM, a profile that was consistent over time. Although the PTM decreased during the study period, the trend over the last two and a half years has been stable.

In the present study, by using appropriate spatial analysis techniques, we showed an association between teenage pregnancy and sociodemographic aspects. In the modeling, the Moran's index identified the presence of autocorrelation in the data, and this component was appropriately incorporated. The final model presented normal residuals, without pattern and spatial autocorrelation^29^.

The association found between teenage pregnancy and socioeconomic factors corroborates the results of national^11,15,17,18^ and international^32,33^ ecological studies, whose authors used different analysis techniques. This study fills a gap in the analysis of individual vulnerability related to early pregnancy in areas with different socioeconomic profiles. In addition, we proved that there is a difference in social vulnerability among young adolescents with a full-term pregnancy in different geographic strata: in those with high prevalence, there is a proportionally greater number of teenage mothers with greater vulnerability than in those with low prevalence. This result fosters the idea that, in addition to being interesting to identify priority areas for implementing public policies aimed at reducing the PTM, it may be relevant to consider the particularities of the profile of young women in different locations. Indeed, authors of some studies highlight the need to consider social inequalities as a premise when planning strategies aimed at coping with pregnancy during adolescence^15,18,34^.

Another interesting finding was the reduction, and subsequent stability, of the PTM over time, which is consistent with other research whose authors also found regional, national, and global reductions in recent years^12,18,34^. However, we show that this movement does not occur uniformly in different locations. While the strata with PR≤1 showed reductions in proportions from January/2013 to July/2017, in PR>1 this reduction was later, taking place only as of January/2016. This finding raises new hypotheses for future research such as the possibility that policies to reduce teenage pregnancy do not reach socioeconomically different territories at the same time. The differences identified in intra-municipal spaces highlight the need for public policies to include territorial and socioeconomic specificities, in order to be more effective.

Misinformation and lack of access to sexual and reproductive health initiatives and services are important risk factors for unintentional teenage pregnancy, often present in situations of poverty, low level of education, low income, and households managed by women^35,36^. The occurrence of pregnancy during adolescence can mean an interruption in the school trajectory and, consequently, better insertion in the job market^35^.

In this study we sought to analyze vulnerability related to teenage pregnancy considering the interrelationship of contexts of social vulnerability and at the individual level. Within the ecological context, its greatest intensity was observed in areas with worse socioeconomic indices, reflecting environments with restricted opportunities regarding living conditions, access to educational and health services and, therefore, development opportunities for these young women. At the individual level, a vulnerability index was developed that, in the extreme, added unfavorable situations — such as repeated pregnancy in adolescence, low level of education, and difficult access measured by the late start of prenatal care. The analysis of these two dimensions showed that there is a greater frequency of more vulnerable adolescent mothers individually in the high prevalence stratum, a situation that can perpetuate the reproduction of social and gender inequalities. Authors of the *Nascer no Brasil* [Birth in Brazil] survey found important inequalities in prenatal care for pregnant teenagers, with a higher concentration of inadequate education, multiparous women, fewer appointments and the beginning of prenatal care in social classes D/E^37^.

Among the limitations, we highlight the use of secondary data, therefore subject to filling errors. Moreover, the data refer to full-term pregnancies and resulted in the registration of a LBC, and it is not possible to consider interrupted pregnancies, regardless of the cause. Vulnerability is a complex phenomenon, which involves multiple determinations associated with individual, social, and programmatic/institutional variables^38^. The index developed to measure vulnerability was based on three aspects only, depending on the availability of data from Sinasc. However, the choice of components was based on studies according to which education, multiparity, and access to health services are associated with groups more prone to vulnerable situations. Mostly, the recurrence of teenage pregnancy takes place in contexts of social inequality^39^. Repetition of this event can lead to failure and school dropout^40^, which is a factor in worsening socioeconomic conditions, restricting the possibilities of professional qualification and insertion in the job market, causing financial dependence and compromising the individual's autonomy. Moreover, researchers show that late initiation of prenatal care was more frequent among black/brown and poor adolescents^37^, compromising the opportunity to anticipate risk identification and prevent complications. The use of robust spatial and temporal analysis techniques also stands out. The absence of similar studies for comparison highlights the relevance of the results found based on the unprecedented use of such techniques in intra-municipal analyses of teenage pregnancy.

All in all, the identification of spatial strata with high and low prevalence of teenage pregnancy highlights its non-random occurrence in space, but depends on socioeconomic factors that are also correlated in space.

The association of the phenomenon with socioeconomic factors demonstrated that, when considering spatial dependence, these have a significant influence on the proportion of pregnant teenagers in different spaces. There was also an association between individual vulnerability and strata with different prevalence of the phenomenon, in addition to the existence of disparities in the reduction in the proportion of teenage mothers in strata with different prevalence over time.

Teenage pregnancy is a complex phenomenon and involves multiple dimensions of human life, related to contexts of socio-cultural and economic vulnerability, encompassing issues of social, ethnic-racial, and gender inequalities. Addressing it requires efforts from different public sectors and civil society, supported by comprehensive protection guidelines and, consequently, the protection of the physical, psychological, and social condition of adolescents, aiming to guarantee their rights as citizens^41^.

We suggest that more studies that consider spatial dependence in the analysis of pregnancy in adolescents be carried out, in order to better understand its relationship with the space in which young women live and their socioeconomic factors. Furthermore, when observing that, even at the intra-municipal level, there are divergences in the distribution of teenage mothers, their individual vulnerabilities, and the way in which the proportion decreases in different prevalence strata, we suggested that such particularities be considered in the formulation of public policies to reducing the occurrence of the phenomenon, including at the intra-municipal level.
