# Supplementary material for: Socioeconomic factors increase the risk of teenage pregnancy: spatial and temporal analysis in a Brazilian municipality
Source: Rev Bras Epidemiol. 2024 Jul 29;27:e240040. doi: 10.1590/1980-549720240040 (PMC11290768; doi:10.1590/1980-549720240040)
Supplement: Translation

# Translation: Fatores socioeconômicos aumentam o risco de gravidez na adolescência: análise espacial e temporal em um município brasileiro

**DOI:** 10.1590/1980-549720240040.2

**Authors:** Camila Meireles Fernandes, Gleice Margarete de Souza Conceição, Zilda Pereira da Silva, Fernando Kenji Nampo, Francisco Chiaravalloti

**Affiliations:** I Universidade de São Paulo, Faculdade de Saúde Pública, Departamento de Epidemiologia – São Paulo (SP), Brasil.; II Universidade Federal da Integração Latino-Americana, Instituto Latino-Americano de Ciências da Vida e da Natureza – Foz do Iguaçu (PR), Brasil.

**Keywords:** Análise espacial, Gravidez na adolescência, Análise de regressão, Distribuição temporal, Fatores socioeconômicos

## Abstract

**Objetivo:**

Avaliar a distribuição da proporção de mães adolescentes (PMA) no tempo e espaço e sua relação com indicadores socioeconômicos e vulnerabilidade social.

**Métodos:**

Realizou-se estudo ecológico com mães adolescentes residentes em 322 setores censitários de Foz do Iguaçu (PR) entre 2013 e 2019. Aglomerados espaciais de mães adolescentes foram identificados por varredura espacial e agrupados em estratos com diferentes prevalências. Avaliou-se a associação entre esses estratos e a vulnerabilidade social individual das mães pelo teste qui-quadrado de Pearson. Ajustaram-se modelos de regressão linear para avaliar associação entre a PMA e fatores socioeconômicos por setor censitário e tendência temporal da PMA nos diferentes estratos.

**Resultados:**

Identificaram-se cinco aglomerados de alta prevalência nas regiões periféricas e seis de baixa na região central do município. Proporcionalmente houve mais mães adolescentes com pior índice de vulnerabilidade no estrato de alta prevalência do que no de baixa. Locais com piores condições socioeconômicas apresentaram maior PMA, perfil que não mudou ao longo do tempo. Para o aumento de uma unidade do Índice Brasileiro de Privação e da proporção de mulheres responsáveis por domicílio, a PMA aumentou respectivamente 3,8 (IC95% 3,1–4,4) e 0,086% (IC95% 0,03–0,14). Houve uma redução na PMA global em parte do período, que ocorreu mais tardiamente nos estratos de maior prevalência, mas as proporções voltaram a ficar estáveis nos últimos anos de estudo.

**Conclusão:**

A gravidez na adolescência se concentra em regiões de pior condição socioeconômica e com maior vulnerabilidade materna e seu comportamento no tempo ocorreu, nessas áreas, de modo distinto.

## INTRODUÇÃO

A adolescência é marcada por mudanças fisiomorfológicas, adaptações psicossociais, sendo determinante para a saúde e bem-estar na vida adulta^1^. A saúde sexual dos adolescentes é parte de seu desenvolvimento, contudo se depara com carência de informações e educação sexual, coerção sexual e violência do parceiro, acesso dificultado aos métodos contraceptivos e abortivos e alta taxa de gravidez^2^.

Mundialmente, a mortalidade materna em adolescentes foi 36,8% maior quando comparada às mulheres de 20 a 24 anos em 2011^3^. Adolescentes grávidas apresentam maiores taxas de depressão e maior uso de substâncias psicoativas quando comparadas a adultas em estudos com dados de 2009 a 2014 no Canadá^4^. Observam-se maiores prevalências de parto prematuro, eclâmpsia, hemorragia pós-parto, recém-nascidos com baixo peso, baixo Apgar e anemia, bem como pior saúde mental no puerpério em estudos até 2020^5–7^.

Apesar da queda global da gravidez na adolescência nas últimas décadas, países da África Subsaariana e América Latina e Caribe continuam com elevadas taxas, 104 e 63 nascimentos por mil adolescentes, respectivamente^8^. O Brasil apresentou redução do percentual de nascidos vivos (NV) de mães adolescentes, contudo apresenta valores elevados, especialmente nas regiões Norte e Nordeste^9^.

A gravidez na adolescência é um desafio, principalmente em países em desenvolvimento, onde políticas de saúde e educação sexual são deficientes ou escassas^10^. Isto afeta, majoritariamente, os indivíduos em vulnerabilidade, tendo como fatores associados, a pobreza extrema, evasão escolar e ser filho/filha de mãe adolescente^5,10^. Envolve fatores socioeconômicos, educacionais, ambientais e comportamentais e não ocorre uniformemente em diferentes regiões e populações, portanto considerar o tempo e o espaço ajudam a otimizar ações preventivas^11,12^.

A partir de 1980, a área da saúde começou a utilizar abordagens espaciais para análise de eventos, rompendo um padrão predominante de estudos com foco só nos indivíduos. Estudos ecológicos, nos quais a unidade de análise corresponde a um agrupamento populacional no tempo e(ou) espaço^13^, ajudam a compreender padrões de doenças e seus desfechos^13,14^.

A maioria dos estudos ecológicos realizados no Brasil analisando espacialmente a gravidez na adolescência teve como unidade de análise os municípios e evidenciam sua associação com fatores socioeconômicos, como escolaridade, índices de Gini e de desenvolvimento humano municipal (IDHM), densidade de moradores por domicílio, renda familiar *per capita* e proporção de população de baixa renda^11,15–19^. Apenas três estudos foram realizados com informações intramunicipais^12,16,20^. Dentre esses estudos, somente alguns^11,15,17,19^ levaram em conta a dependência espacial do fenômeno estudado, produzindo resultados mais acurados.

Apesar da importância da gravidez na adolescência na saúde pública^9,17^, poucas pesquisas analisaram-na espacialmente, evidenciando uma lacuna na área, especialmente quando se trata de avaliações intramunicipais. Dado que as técnicas de análise espacial estão em constante atualização, novos estudos da gravidez na adolescência com enfoque espacial podem subsidiar a tomada de decisão por parte de gestores e contribuir para o levantamento de hipóteses tendo como premissa a importância do território e fatores socioeconômicos na ocorrência do fenômeno.

Este estudo se propôs a avaliar a distribuição da proporção de mães adolescentes (PMA) no tempo e no espaço e sua relação com indicadores socioeconômicos e vulnerabilidade social no município de Foz do Iguaçu, entre 2013 e 2019.

## MÉTODOS

Trata-se de um estudo ecológico.

### Caracterização do local de estudo

Foz do Iguaçu se localiza no Paraná, Brasil, fazendo divisa com Paraguai e Argentina. Em 2019, possuía uma população de 258.532 habitantes e alto produto interno bruto (PIB) *per capita* (R$ 60.997,41). A taxa de mortalidade infantil registrou decréscimo no período 2023–2019 (de 14,14 para 10,17 óbitos por mil NV) e o IDHM evoluiu de médio (0,663), em 2000, para alto, em 2010 (0,751)^21^. Embora esses indicadores apontem para seu alto desenvolvimento em relação ao país, o Índice Brasileiro de Privação (IBP-Cidacs) evidencia desigualdades intramunicipais. Em 2010, o IBP variou de −3,04 a 2,99 nos diferentes setores censitários (SC) urbanos. Esse é um indicador de situação socioeconômica desenvolvido com base em indicadores de renda, escolaridade e condição dos domicílios, utilizando dados do Censo de 2010. Quanto maior o seu valor, maior a privação^22^.

### Dados

Os dados sobre os NV, entre janeiro/2013 e dezembro/2019, de mães residentes no município foram obtidos do Sistema de Informação Sobre Nascidos Vivos (Sinasc). Consideraram-se as informações das declarações de nascidos vivos (DNV): idade, endereço, data do parto, número de gestações anteriores, escolaridade e início do pré-natal. Foram excluídos registros sem endereço completo, impossibilitando a geocodificação. Nas gestações múltiplas, apenas um registro foi mantido para evitar duplicações.

Na análise espacial, os endereços das mães foram convertidos em coordenadas geográficas, por meio do Google Meus Mapas^23^. Elas foram exportadas para o *software* QGIS^24^ e sobrepostas ao mapa contando com 322 SC, já considerando a exclusão de dois setores periféricos e sem nascimentos e um setor mesclado a um vizinho, por conter apenas um nascido e de mãe adolescente (resultando em uma proporção de 100%). A comparação desse com o dos SC do município^25^ possibilitou a contagem de mães adolescentes e do total de mães em cada setor, obtendo-se a PMA, definida como o quociente entre o número de mães adolescentes e o total de mães multiplicado por 100. Para a análise temporal, foi calculada a PMA mensal.

### Análise estatística

#### Identificação de aglomerados espaciais

Para a identificação de aglomerados espaciais de alta e baixa prevalência de gravidez na adolescência, utilizou-se o *software* de varredura SaTScan, assumindo distribuição de Poisson^26^. Constituíram-se bancos de dados com as informações do número de mães adolescentes, do total de mães e das coordenadas dos centroides dos setores. O tamanho máximo da população de mães para a janela de varredura foi determinado utilizando o índice de Gini^27^. O p-valor foi obtido por meio do método de Monte Carlo. Os resultados foram agregados aos setores no QGIS, obtendo-se o mapa com os aglomerados e respectivas razões de prevalências (RP).

Com base nesses resultados, os setores foram agrupados em três estratos, compondo áreas não necessariamente contíguas: "RP>1" ou "alta prevalência", contendo os setores pertencente aos aglomerados com RP significativamente maior do que 1; "RP=1", com os que não pertenciam a nenhum aglomerado; "RP<1" ou "baixa prevalência", com os pertencentes aos aglomerados com RP significativamente menor do que 1.

#### Associação entre tipo de estrato e vulnerabilidade da mãe

A Organização Mundial de Saúde (OMS) aponta que o progresso na redução da gestação na adolescência tem sido mais lento entre grupos vulneráveis, levando a uma crescente desigualdade. Com dados do Sinasc, construiu-se um índice de vulnerabilidade para adolescentes, composto por três variáveis assumindo os valores 0 ou 1: gestações anteriores (0, se nenhuma; 1, se uma ou mais), escolaridade (0, se cursou o ensino médio ou mais; 1, se cursou o ensino fundamental II completo ou menos) e início do pré-natal (0, se teve início no primeiro trimestre; 1, se teve início no segundo trimestre ou depois). A soma dos valores atribuídos a cada mãe produziu um índice variando de 0 a 3: quanto maior o seu valor, maior a sua vulnerabilidade social. Para avaliar se esse índice estava associado ao estrato de residência, realizou-se o teste de χ^2^ de Pearson.

#### Modelagem espacial

Para avaliar se a gravidez na adolescência estava associada ao nível socioeconômico da área de residência, foi ajustado um modelo de regressão linear múltipla, tendo como variável resposta a PMA e, como variáveis independentes, o IBP e a proporção de mulheres responsáveis pelo domicílio (PMRD), obtida com base em dados do Censo de 2010^28^, segundo o setor. Previamente, realizou-se uma análise exploratória dos dados. Foi utilizado o teste de Moran Global para avaliar a autocorrelação espacial da PMA e dos resíduos dos modelos obtidos. Considerou-se a matriz de vizinhança por contiguidade Queen. Inicialmente, foi ajustado um modelo de regressão linear clássico e identificada dependência espacial dos resíduos. Após, foi utilizado o teste do multiplicador de Lagrange, que mostrou que o modelo espacial autorregressivo (SAR) mais adequado para representar a autocorrelação espacial não explicada pelo modelo inicial foi o da defasagem espacial^29^.

#### Modelagem temporal

Para avaliar a tendência da proporção de gravidez na adolescência, ajustou-se um modelo de regressão linear múltipla. A variável resposta foi a PMA mensal e as explicativas foram o tempo (meses) e o estrato de residência. Para avaliar sua suposição de normalidade, utilizou-se o teste de Kolmogorov-Smirnov. Para modelar mudanças nas tendências durante o período, utilizaram-se técnicas de regressão segmentada com dois pontos de inflexão sugeridos pelos dados: janeiro/2016 e julho/2017. Um intercepto e uma inclinação foram estimados para cada um dos três períodos determinados (jan./2013–jan./2016, jan./2016–jul./2017, jul./2017–dez./2019), segundo o estrato. Para avaliar se a tendência em determinado estrato era a mesma nos três períodos, bem como se a tendência em um determinado período era a mesma nos três estratos, construíram-se contrastes, sob a hipótese linear geral^30^ envolvendo as inclinações correspondentes. Nas situações em que elas não foram significativamente diferentes, uma única inclinação foi ajustada. Com base no modelo final, apresentaram-se as PMA ajustadas no início e no final de cada período, além da variação desses percentuais no tempo, segundo o estrato. Para avaliar se o modelo em questão estava bem ajustado, realizou-se uma análise de resíduos.

Com exceção da análise de varredura, as demais realizaram-se no software R^31^. O nível de significância dos testes foi fixado em 5%.

Esta pesquisa foi aprovada pelo Comitê de Ética em Pesquisa do Centro Universitário Dinâmica das Cataratas (UDC), CAAE 33186620.7.0000.8527, em 31 de julho de 2020.

## RESULTADOS

Após a aplicação dos critérios de exclusão, o número total de mães foi 29.507; dessas, 4.117 (13,95%) eram adolescentes. Excluíram-se registros referentes a partos gemelares (337 mães, sendo 7,71% adolescentes) e sem endereço completo (809 mães, sendo 19,03% adolescentes).

### Aglomerados espaciais e associação com vulnerabilidade da mãe

O tamanho máximo da população de mães para a janela de varredura estimada pelo índice de Gini foi 6%. A Figura 1A apresenta os 11 aglomerados identificados, sendo cinco de alta prevalência (45 setores) e seis de baixa (83 setores).

**Figura 1:** Aglomerados espaciais de alta e baixa prevalência de mães adolescentes (A), Proporção de mães adolescentes (B), Índice brasileiro de privação na área urbana (C) e Proporção de mulheres responsáveis por domicílio (D), segundo setor censitário. Foz do Iguaçu (PR), Brasil, 2013–2019.

Na Tabela 1, observa-se associação entre o índice de vulnerabilidade e os dois estratos de setores formados pelos aglomerados de alta e baixa prevalência (p<0,001). À medida que aumenta o índice de vulnerabilidade, aumenta a proporção de mães com RP>1 e diminui a proporção com RP=1 e RP<1. Proporcionalmente, há mais mães adolescentes com pior índice de vulnerabilidade no estrato de alta prevalência do que no de baixa.

**Tabela 1:** Distribuição de mães adolescentes segundo índice de vulnerabilidade social e tipo de aglomerado espacial. Foz do Iguaçu (PR), Brasil, 2013–2019.

Índice de vulnerabilidade	Tipo de aglomerado						Total		p-valor
	RP>1		RP=1		RP<1				
	n	%	n	%	n	%	n	%	
0	344	24,33	874	61,81	196	13,86	1.414	100	<0,001
1	447	27,63	964	59,58	207	12,79	1.618	100	
2	289	33,45	487	56,37	88	10,19	864	100	
3	77	35,48	114	52,53	26	11,98	217	100	
Total	1.157	28,13	2.439	59,3	517	12,57	4.113	100	

RP: razões de prevalências.

### Modelagem espacial

O teste de Moran apontou a existência de autocorrelação espacial nos resíduos (I=0,059, p=0,025) do modelo de regressão clássico, e um modelo de defasagem espacial foi utilizado. Esse mostrou associação direta da PMA com o IBP e a PMRD (Figura 2): a cada aumento de uma unidade no IBP, a PMA aumenta 3,8% (IC95% 3,1–4,4) e, a cada aumento de 1% na PMRD, a PMA aumenta 0,086% (IC95% 0,03–0,14), em média. Essas associações se refletem nas Figuras 1B, 1C e 1D, respectivamente com a distribuição geográfica da PMA, IBP e PMRD. O coeficiente espacial autorregressivo^29^ (Rho=0,14 p=0,055) representou a dependência espacial não explicada pelas covariáveis incluídas no modelo. O coeficiente de determinação foi 0,44, isto é, o modelo explica 44% da variabilidade da PMA. Esse modelo apresentou menor AIC (2055,8) do que o modelo clássico (2057,4), e seus resíduos apresentaram normalidade (p=0,05343, no teste de Kolmogorov-Smirnov) e bom ajuste.

**Figura 2:** Diagrama de dispersão da proporção de mães adolescentes em função do índice brasileiro de provação e da proporção de mulheres responsáveis pelo domicílio. Foz do Iguaçu (PR), Brasil, 2013–2019.

### Modelagem temporal

A Figura 3A apresenta a PMA ao longo dos anos, evidenciando decréscimo entre 2013 e 2019, variando de 17,67% para 12,13%. A Figura 3B apresenta as PMA observadas (representados por pontos) e ajustadas (representados por retas) ao longo dos meses, segundo o estrato de residência. A Tabela 2 apresenta os valores ajustados para as proporções no início e final de cada período e a variação estimada ao longo do tempo para cada estrato.

**Figura 3:** Proporção de mães adolescentes ao longo dos anos (A) e dos meses, segundo a razão de prevalência dos estratos (B). Foz do Iguaçu (PR), Brasil, 2013–2019.

**Tabela 2:** Percentual de mães adolescentes ajustado no início e no final de cada período e variação estimada da proporção ao longo do tempo, segundo estrato. Foz do Iguaçu (PR), Brasil, 2013–2019.

RP do estrato	Proporção ajustada em jan./2013	Variação no período jan./2013–jan./2016	Proporção ajustada em jan./2016	Variação no período jan./2016–jul./2017	Proporção ajustada em jul./2017	Variação no período jul./2017–dez./2019	Proporção ajustada em dez/2019
RP<1	10,9 (9,4; 12,3)	−0,10* ↓ (−0,14; −0,06)	7,3 (6,4; 8,1)	−0,10* ↓ (−0,14; −0,06)	5,5 (4,3; 6,6)	0,04 → (−0,04; 0,12)	6,7 (4,8; 8,7)
RP=1	17,8 (16,3; 19,2)	−0,10* ↓ (−0,14; −0,06)	14,1 (13,2; 15,1)	−0,10* ↓ (−0,14; −0,06)	12,3 (11,1; 13,6)	−0,02 → (−0,12; 0,08)	11,8 (9,5; 14,1)
RP>1	24,2 (22,0; 26,4)	0,03* → (−0,06; 0,13)	25,3 (23,4; 27,1)	−0,34* ↓ (−0,49; −0,19)	19,2 (17,6; 20,7)	0,04 → (−0,04; 0,12)	20,4 (18,7; 22,2)

RP: razões de prevalências.

* p<0,001. As setas indicam a variação temporal no período em específico. A seta para baixo indica redução, e a seta para o lado direito indica que não houve variação no período.

Nos estratos RP<1 e RP=1, a PMA diminuiu 0,1% (IC95% 0,06–0,14) ao mês até julho de 2017. No primeiro, a proporção passou de 10,9% (em janeiro/2013) para 5,5% (em julho/2017). No segundo, a proporção passou de 17,8% (em janeiro/2013) para 12,3% (em julho/2017). A partir de julho/2017, as tendências não foram significativas. Em dezembro/2019, as proporções ajustadas foram 6,7 e 11,8% nos estratos RP<1 e RP=1, respectivamente.

Ao contrário dos outros dois estratos, no RP>1, a proporção só passou a diminuir em janeiro/2016. De janeiro/2016 a julho/2017, houve queda estimada em 34% (IC95% 19–49) ao mês, expressivamente maior do que aquela observada nos outros estratos. A partir de julho/2017, a exemplo do que aconteceu nos outros estratos, a queda cessou e a tendência permaneceu constante até o final do estudo.

Durante todo o período, as PMA ajustadas foram maiores no estrato RP>1, seguido do RP=1 e, por último, no RP<1. Em janeiro/2013, a proporção ajustada no estrato RP>1 era mais do que o dobro da proporção no RP<1; em julho/2017, este valor chegou a 3,5 vezes e não sofreu grandes alterações até o final do estudo. Os resíduos da modelagem temporal apresentaram normalidade (p=0,3525, no teste Kolmogorov-Smirnov) e bom ajuste.

## DISCUSSÃO

As análises realizadas mostram uma relação consistente da ocorrência de gravidez na adolescência com indicadores socioeconômicos e vulnerabilidade social. Locais com maior índice de vulnerabilidade da mãe e piores condições socioeconômicas apresentaram maior PMA, perfil consistente no tempo. Apesar de a PMA diminuir no período de estudo, a tendência nos últimos dois anos e meio foi de estabilidade.

O presente estudo, utilizando técnicas adequadas de análise espacial, evidenciou associação da gravidez na adolescência e aspectos sociodemográficos. Na modelagem, o índice de Moran identificou a presença de autocorrelação dos dados, e esse componente foi adequadamente incorporado. O modelo final apresentou resíduos normais, e sem padrão e autocorrelação espacial^29^.

A associação encontrada entre gestação na adolescência e fatores socioeconômicos corroboram os resultados de estudos ecológicos nacionais^11,15,17,18^ e internacionais^32,33^, que utilizaram diferentes técnicas de análise. Este estudo preenche uma lacuna da análise da vulnerabilidade individual relacionada à gestação precoce em áreas com diferentes perfis socioeconômicos. Também comprovou que há diferença de vulnerabilidade social entre as jovens adolescentes que levam a gestação a termo em diferentes estratos geográficos: nos de alta prevalência, há proporcionalmente um número maior de mães adolescentes com maior vulnerabilidade do que nos de baixa. Esse resultado fomenta a ideia de que, além de ser interessante a identificação de áreas prioritárias para execução de políticas públicas visando à redução da PMA, pode ser relevante que se considerem as particularidades do perfil das jovens nas diferentes localidades. De fato, algumas pesquisas ressaltam a necessidade de considerar as desigualdades sociais como premissa no planejamento de estratégias voltadas ao enfrentamento da gestação durante a adolescência^15,18,34^.

Outro achado interessante foi a redução, e a posterior estabilidade, da PMA no tempo, que é condizente com outras pesquisas que também verificaram reduções regionais, nacionais e globais nos últimos anos^12,18,34^. Entretanto este estudo mostra que esse movimento não ocorre do mesmo modo nos diferentes locais. Enquanto os estratos com RP≤1 apresentaram reduções nas proporções de janeiro/2013 a julho/2017, no RP>1 essa redução foi mais tardia, acontecendo somente a partir de janeiro/2016. Tal achado levanta novas hipóteses para futuras pesquisas como a possibilidade de as políticas para redução da gravidez na adolescência não atingirem ao mesmo tempo territórios socioeconomicamente diferentes. As diferenças identificadas nos espaços intramunicipais destacam a necessidade de as políticas públicas incluírem especificidades territoriais e socioeconômicas, de modo a serem mais efetivas.

A desinformação e a falta de acesso às ações e serviços de saúde sexual e reprodutiva são importantes fatores de risco para a gravidez não intencional na adolescência, muitas vezes presentes em situações de pobreza, baixa escolaridade, baixa renda e domicílios com responsáveis mulheres^35,36^. A ocorrência da gestação na adolescência pode significar a interrupção na trajetória escolar e, consequentemente, de melhor inserção no mercado de trabalho^35^.

Este estudo procurou analisar a vulnerabilidade relacionada à gravidez na adolescência considerando a interrelação de contextos de vulnerabilidade social e no nível individual. No contexto ecológico, observou-se sua maior intensidade em áreas com piores índices socioeconômicos, expressando ambientes com oportunidades restritas nas condições de vida, no acesso a serviços educacionais e de saúde e, portanto, nas oportunidades de desenvolvimento para essas jovens. No nível individual, foi produzido um índice de vulnerabilidade que no seu extremo agregou situações desfavoráveis como repetição de gestação na adolescência, baixa escolaridade e dificuldade de acesso medido pelo início tardio do pré-natal. A análise dessas duas dimensões mostrou que há maior frequência de mães adolescentes mais vulneráveis individualmente no estrato de alta prevalência, situação que pode perpetuar a reprodução das desigualdades sociais e de gênero. A pesquisa Nascer no Brasil, encontrou importantes desigualdades na assistência pré-natal de gestantes adolescentes, com maior concentração de escolaridade inadequada, multíparas, menor número de consultas e inicio do pré-natal nas classes D/E^37^.

Dentre as limitações, destaca-se a utilização de dados secundários, portanto sujeitos a erros de preenchimento. Adicionalmente os dados se referem a gestações que foram levadas a termo e resultaram no registro de uma DNV, não sendo possível considerar as gestações interrompidas, independente da causa. A vulnerabilidade é um fenômeno complexo, que envolve múltiplas determinações associadas a variáveis de cunho individual, social e programático/institucional^38^. O índice criado para mensurar a vulnerabilidade foi baseado em apenas três aspectos, condicionando à disponibilidade de dados do Sinasc. Entretanto a escolha dos componentes se baseou em estudos que mostram que escolaridade, multiparidade e acesso a serviços de saúde estão associados com grupos mais sujeitos a situações vulneráveis. Na maioria das vezes, a reincidência de gravidez na adolescência ocorre em contextos de desigualdade social^39^. A repetição desse evento pode levar à reprovação e ao abandono escolar^40^, que é um fator para agravamento das condições socioeconômicas, restringindo as possibilidades de qualificação profissional e inserção no mercado de trabalho, provocando dependência financeira e comprometendo sua autonomia. Adicionalmente, estudos mostram que o início tardio do pré-natal foi mais frequente entre adolescentes pretas/pardas e pobres^37^, comprometendo a oportunidade de antecipar a identificação de riscos e prevenir complicações. Como outro ponto forte, destaca-se o uso de técnicas robustas de análise espacial e temporal. A ausência de estudos similares para comparação ressalta a relevância dos resultados encontrados com base no uso inédito de tais técnicas em análises intramunicipais de gestação na adolescência.

Em conclusão, a identificação de estratos espaciais de alta e baixa prevalência de gravidez na adolescência evidencia sua ocorrência não aleatória no espaço, mas depende de fatores socioeconômicos também correlacionados no espaço.

A associação do fenômeno com fatores socioeconômicos, demonstrou que, ao considerar a dependência espacial, esses apresentam expressiva influência na proporção de gestantes adolescentes em diferentes espaços. Também se verificou associação entre vulnerabilidade individual e estratos de diferentes prevalências da ocorrência do fenômeno, além da existência de disparidades na redução da proporção de mães adolescentes em estratos de diferentes prevalências temporalmente.

A gravidez na adolescência é um fenômeno complexo e envolve múltiplas dimensões da vida humana, relacionadas a contextos de vulnerabilidade sociocultural e econômica, perpassando por questões de desigualdades sociais, étnico-raciais e de gênero. O seu enfrentamento exige esforços de distintos setores públicos e da sociedade civil, amparados nas diretrizes de proteção integral e consequentemente a proteção da condição física, psíquica e social dos adolescentes, visando garantir seus direitos de cidadãos^41^.

Sugere-se que mais estudos que considerem a dependência espacial na análise da gestação em adolescentes sejam elaborados, a fim de entender melhor a sua relação com o espaço no qual as jovens habitam e seus fatores socioeconômicos. Além disso, ao observar que, mesmo em nível intramunicipal, existem divergências na distribuição das mães adolescentes, suas vulnerabilidades individuais e o modo como a proporção diminui em diferentes estratos de prevalência, sugere-se que tais particularidades sejam consideradas na elaboração de políticas públicas para redução do fenômeno, inclusive em nível intramunicipal.
